# Interdisciplinary recognizing and managing of drug‐induced tardive oromandibular dystonia: two case reports

**DOI:** 10.1002/ccr3.1548

**Published:** 2018-09-26

**Authors:** Merete Bakke, Tove Henriksen, Heidi Bryde Biernat, Torben Dalager, Eigild Møller

**Affiliations:** ^1^ Department of Odontology Faculty of Medical and Health Sciences University of Copenhagen Copenhagen Denmark; ^2^ Department of Neurology and Clinical Neurophysiology (Dystonia Clinic) Bispebjerg University Hospital University of Copenhagen Copenhagen Denmark

**Keywords:** Antipsychotics, botulinum toxin, bruxism, masticatory muscles, tardive dystonia

## Abstract

Tardive dystonia is a risk factor in medical antipsychotic treatment. It often begins with repetitive involuntary jaw and tongue movements resulting in impaired chewing and detrimental effect on the dentition. The orofacial dysfunction may go unrecognized in a neurological setting. The diagnosis may be difficult so we suggest interdisciplinary collaboration.

## Background

Dystonia is characterized by abnormal involuntary movements of muscles (WHO ICD‐10 G24). The disease can be divided into idiopathic, genetic, and acquired, that is, resulting from environmental or other damage to the brain or from exposure to dopamine receptor blocking agents such as antipsychotics, antitussive drugs, and antiemetics. However, the pathophysiology of TD is unclear.

The acute drug‐induced dystonia develops days or weeks after start of the antipsychotic treatment 1. Characteristic signs are acute affection of the ocular muscles with prolonged deviation of the eyes in an upward direction (oculogyric crises), stiffness in the neck and shoulder, and tongue protrusion. It often ceases if the medications are stopped quickly. Drug‐induced dystonia with a delayed onset also referred to as tardive dyskinesia (TD), usually begins in the throat, face, and masticatory muscles as a form of oromandibular dystonia (OMD) with repetitive, complex chewing motions, but other tardive syndromes such as drug‐induced chorea may also appear. It may also spread to the whole body even if the medication is stopped 2.

The forceful contractions and persistent repetitive dystonic movements are associated with difficult opening or closing of the mouth and affect chewing, swallowing, and speech 3, 4. It may also cause bruxism during wakefulness with excessive teeth grinding and jaw clenching that result in tooth wear and damage of dental restorations. Making the diagnosis is sometimes not so easy. Patients may be misdiagnosed as having similar and perhaps related disorders such as Parkinson's disease, essential tremor, Tourette's syndrome, temporomandibular disorders, nocturnal bruxism, and even primary denture problems. So there are many differential diagnoses. Consequently, the diagnostic delay is often significant. Multidisciplinary collaboration with exchange of knowledge is therefore advisable. Efficient diagnosis may also require electromyographic and dental examinations as well as paraclinical tests in terms of scannings and blood tests.

In patients receiving antipsychotic medication, the incidence over a 4‐year period is approximately 20% with highest incidence in middle‐aged and elderly patients, particularly women. To prevent TD, the lowest possible dose should be used, when antipsychotics are needed and second‐generation antipsychotics should probably be preferred 5. Intramuscular injections with botulinum toxin (BTX) and deep brain stimulation may be helpful for decreasing TD 6.

## Patients and Methods

The characteristics and treatment are described in two elderly women, Patients A and B, with persistent drug‐induced TD based on history, clinical, video and electromyographic recordings, blood tests, and imaging at the Department of Neurology and Clinical Neurophysiology, Department of Clinical Biochemistry, the Department of Radiology and Imaging in the Bispebjerg University Hospital, and the Department of Odontology, University of Copenhagen. Written informed consent was obtained to publish their data extracted from the electronic records in the departments in an anonymized form.

**Figure 1:**
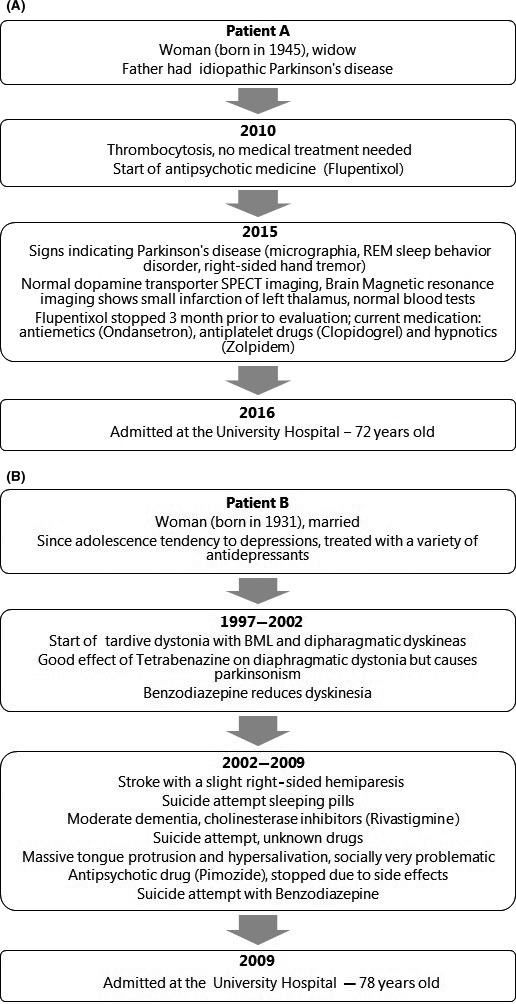
(A) Medical history for Patient A prior to the referral to the University Hospital. (B) Medical history for Patient B prior to the referral to the University Hospital.

As shown in Figure 1A and B, their medical history included a variety of drugs prior to referral, including antipsychotic and antidepressant treatment, which may induce TD. The data on their previous medication are partly obtained retrospectively. The content in the figures is based on the available information on their drug consumption during the period of respectively 6 (Patient A) and more than 60 years (Patient B).

### Patient A

#### Referral

A woman born in 1945 referred for constant munching and biting. Besides the long‐term use of antipsychotic medicine, her medical history also included genetically predisposition and signs of Parkinson's disease (Fig. 1A), which could be relevant for diagnosis of her movement disorder.

#### Status at the time of referral to the hospital (2016)

Socially handicapped and extremely bothered by reduced eye blinking, oromandibular, mainly biting movements, oligomimia, intermittent right‐sided resting tremor enhanced by mirror movements, and slight rigidity at the right side. MR scanning showed sequelae from left‐sided thalamus infarction, which could explain some of her symptoms. Current medication was antiepileptics (Clonazepam and Gabapentin), antidepressants and antianxiety (Fluoxetine and Diazepam), antiplatelet drugs (Clopidogrel), hypnotics (Zolpidem), and analgesics (Paracetamol and Codeine).

### Clinical follow**‐**up, scannings, further diagnostics, and treatment

Initially, there was good effect from the current medication on the orofacial dyskinesia but increasing parkinsonism and problems with the gait. Findings in DAT‐SPECT scannings in 2015 and 2016 were normal and symmetrical and could not support the diagnosis of Parkinson's disease. Higher doses of Diazepam decreased the parkinsonism. Hence, the clinical status showed only slurred speech and bucco‐linguo‐masticatory (BLM) syndrome with jaw and lip protrusion and vermicular tongue movements. Increased doses of Clonazepam reduced the BLM syndrome slightly. As her jaw function was impaired, she was referred for oromandibular and dental evaluation.

#### Oromandibular evaluation including electromyography (2017)

Impaired orofacial function with a high impact on daily life (Table 1) with constant bruxing and munching movements at a frequency of ½–1 Hz, often chewing of cheeks and tongue, and local fatigue and tenderness. The subjective visual analog scale (VAS) for the impact of OMD on daily life was 9 (0–10), and the Fahn–Marsden Dystonia Rating Scale (F‐M DRS for mouth) 8 (0–8). The involuntary movements were reduced when speaking or chewing, and there were no reports of sleep bruxism. The chewing was slow and difficult partly because of hyposalivation. The salivary low secretion rate was probably related to the drug intake. Quantitative electromyographic (EMG) recordings showed very high level of resting activity in masticatory muscles and revealed turns per second higher than 100 in anterior temporal and lateral pterygoid muscles (Table 1) corresponding to her ongoing jaw movements. The range of jaw opening mobility was normal. The patient had full and functional complement of teeth, even if there was heavy tooth wear and damages in the restorations related to the dystonia. It was concluded that the functional problems were caused by the dystonic activity and not related to the dental state.

**Table 1:** Patient characteristics

Patient	Oromandibular dystonia				Orofacial function			
	Dominant dystonia type	Impact on daily life (VAS 0–10)^a^	F‐M dystonia rating scale (OMD: 0–8)^b^	Needle electromyography (>100 turns/sec)^c^	Orofacial dysfunction screening (0–12)^d^	Jaw Disability Checklist (RDC/TMD 0–12)^e^	Subjective mastication index (0–4)^f^	Chewing time (apple 95% CI: 10–47 sec)
A	Munching and biting	9	8	Ant. temporalis, Lat. pterygoid	6	3	1	64
B	Tongue protrusion	10	8	Genioglossus	7	4	2	84

a visual analog scale.

b Fahn–Marsden Dystonia Rating Scale.

c Werdelin et al. 12.

d Nordic Orofacial Screening – Test 13.

e Research Diagnostic Criteria for Temporomandibular Disorders 14.

f Chewing ability after Yoshida et al. 15.

#### Additional treatment and diagnosis

As a consequence of the oromandibular findings, intramuscular injections with botulinum toxin were planned to diminish the dystonic bruxing and munching and its detrimental effects. The patient was treated with repeated injections of 25 units IncobotulinumtoxinA (BTX) in the temporalis and lateral pterygoid muscles on each side performed with EMG guidance. Recently, she also complained of severe pain and stiffness in the neck, shoulders, and arms, which her general practitioner considered to be polymyalgia rheumatica. This was later rejected by blood tests and might also be related to left‐sided thalamic infarct.

#### Current status 2017/2018

**Figure 2:**
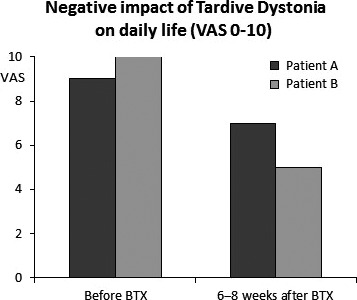
The effect of BTX injections on daily life in two patients with drug‐induced tardive oromandibular dystonia as measured by subjective visual analog scales. Patient A with dystonic biting and bruxing movements was treated with 25 units IncobotulinumtoxinA in the anterior temporal and lateral pterygoid muscles on both sides. Patient B with dystonic jaw opening and repeated tongue protrusion was treated with 15 units IncobotulinumtoxinA in the genioglossus muscles on both sides. All injections were guided by electromyography.

The combined treatment with medication and injections has a reducing, moderate effect on the involuntary movements and the jaw fatigue and tenderness. The OMD impact on daily life was reduced from 9 to 7 1–2 months after injections when the BTX treatment is most effective (Fig. 2). In addition, she also wears a hard acrylic occlusal splint to prevent further dental attrition**.** However, lately her tongue has become clinically hyperactive. Correspondingly, EMG reveals a very high level of resting activity in the genioglossus muscles and turns per second higher than 100. This can be treated with BTX injections but are often associated with side effects.

### Patient B

#### Referral

A woman born in 1931 referred for repeated tongue protrusion. Besides the long‐term use of antidepressant and antipsychotic medicine, her medical history included a minor stroke and three suicide attempts with drugs. In 1997, she developed dystonic movements in her thoracic diaphragm and soon after also in the oromandibular region.

#### Status at the time of referral to the hospital (2009)

Extremely bothered by the involuntary repetitive movements of the tongue in and out of the mouth and drooling. Current medication to reduce the TD was Levetiracetam, Tetrabenazine, and Orphenadrine, which did not reduce the TD sufficiently. Her other medication included Venlafaxine, Alprazolam, Rivastigmine, Asasantin for moderate dementia and her psychiatric and mood disorders, and Atropine for drooling. In general, the effect of the current pharmacological treatment was not sufficient.

#### Oromandibular evaluation including electromyography (2010)

Impaired orofacial function with a high impact on daily life (Table 1) with jaw opening and repeated tongue protrusion every 5 sec, and marked drooling. The subjective visual analog scale (VAS) for the impact of OMD was 10 (0–10) and the Fahn–Marsden Dystonia Rating Scale (for mouth) 8 (0–8). The salivary secretion rate was below average so the sialorrhea was secondary, that is, due to insufficient or infrequent swallowing. Speech was weak, but understandable. Chewing was slow, and tough food was avoided. EMG showed a very high level of resting activity and turns per second higher than 100 in the genioglossus muscles (Table 1) corresponding to the involuntary protrusive tongue movements. There was antagonistic activity in the jaw opening muscles during biting. The range of jaw opening mobility was normal. The patient had a well‐fitting partial denture fixed with braces in the upper jaw and almost full complement of teeth in the lower jaw. Thus, the dental state did not cause the deviant activity although some of the chewing problems could be associated with the partial denture in the maxilla. This type of rehabilitation generally improves the chewing ability, but is not able to fully restore the masticatory function.

#### Additional treatment and further diagnostics

As a consequence of the EMG findings, the patient was treated several times with 15–20 units IncobotulinumtoxinA in the genioglossus muscles in both sides to inhibit the tongue protrusion. A submandibular approach and EMG guidance were used with good effect 7. Two months after BTX treatment, the VAS for OMD was reduced from 10 to 5 (Fig. 2) and the FMDRS for the mouth from 8 to 3. Eventually, the tongue protrusion decreased (to VAS 1 and FMDRS 0), the drooling was reduced, and she stopped the BTX treatment. After a couple of years, parkinsonism emerged. In 2013, Parkinson's disease was diagnosed in addition to the TD on the basis of an abnormal DAT‐SPECT with asymmetrically reduced FP‐CIT uptake in striatum.

#### Current status 2017/2018

She is now treated for Parkinson's disease with some reemerging resting tremor of upper extremities and dystonia. She is mobilized with a walker‐rollator. In addition she is largely anarthric and communicates with gestures. The present medication is Parkinson's medicine (Carbidopa/Levodopa, Benserazide Hydrochloride/Levodopa), antiepileptics (Levetiracetam), anticholinergic (Orphenadrine), antihyperkinetic (Tetrabenazine), antidepressant (Venlafaxine), antidementia (Rivastigmine), hypnotics (Zolpidem). With this medication, the patient indicates further relief of the symptoms.

## Discussion and Conclusion

The cases illustrate the problems of treating patients with psychiatric or mood disorders in an effective way while avoiding side effects. In general, the most frequent drugs causing TD are neuroleptics, such as Benzamides, Thiethylperazine, and calcium‐channel blockers. Both patients have been treated with antipsychotics over a long period of time prior to the emergence of dystonia. Other drugs had also been administered, and other complaints and conditions made the diagnosis complicated. A thorough medical history and a multidisciplinary approach with clinical and paraclinical evaluations such as electromyography, scannings, and blood tests are important to make the diagnosis of TD and to treat it as well as to identify possible differential diagnoses and additional conditions.

Treatment of TD is generally difficult due to limited data and incomplete understanding of the mechanism, and it necessitates a stepwise approach 9. It is also important to evaluate the TD treatment on an individual basis with careful consideration of the severity of symptoms as in the present cases. Optimization of antipsychotic therapy should be considered before initiation of medical treatment of the TD 10. There are no disease‐modifying treatments, but data from recent studies also indicate possible improvements in TD after switching from first‐ to second‐generation antipsychotics. However, there is some evidence of symptomatic treatment with Clonazepam, Ginkgo biloba, Amantadine, and Tetrabenazine (American Academy of Neurology, Guidelines 2013), as well as injections with botulinum toxin 10, 11. If intractable or unresponsive to the array of pharmaceutical interventions, more invasive treatments such as deep brain stimulation may be considered for TD, but the extent of the treatment must always be balanced according to the degree and impact of the symptoms on daily life and orofacial function.

The present reports describe two cases with characteristic features of TD diagnosed and treated by a multidisciplinary team of movement disorder specialists, neurophysiologists, and odontological experts in temporomandibular disorders and orofacial pain 8. However, the cases were not simple. They illustrate diagnostic problems and demonstrate that TD patients may also suffer from disorders with similar symptoms. Therefore, the interdisciplinary cooperation was essential. In both cases, the dystonic activity was reduced by a combination of pharmacologic treatment and BTX injections in the muscles.

In Patient A with dystonic bruxing and munching movements, the condition was more drug‐resistant than in Patient B with dystonic jaw opening and protrusive tongue movements. However, in both cases TD was significantly alleviated but not cured with correction and changes in the drug intake and/or BTX injections in the dystonic muscles. Patient A also had a bite splint to prevent or minimize further attrition and dental trauma. Later, her TD spread to tongue muscles, which will be BTX‐treated as well. In Patient B, the prosthetic reconstruction even well‐fitted might have contributed to the impaired chewing function. She stopped the BTX treatment as the tongue protrusion decreased, but later she developed Parkinson's disease and is treated accordingly.

In conclusion, the two cases with TD show the importance of detailed medical history, specialist examinations, and interdisciplinary collaboration to ensure a correct diagnosis. They also illustrate the significance of following the patient's clinical presentation and symptoms closely over time to tailor the treatment individually as TD may change and can prove hard to treat. Lastly, the report demonstrates the orofacial dysfunction associated with TD, which can easily be overlooked in a neurological setting. This aspect is essential because of its effects on the patients’ well‐being, social activities, and nutrition.

## Conflict of Interest

None declared.

## Authorship

All authors: participated in the patient evaluation and/or treatment prepared the report and have approved the manuscript before submission.
